# Limited range correlations, when modulated by firing rate, can substantially improve neural population coding

**DOI:** 10.1186/1471-2202-16-S1-O16

**Published:** 2015-12-18

**Authors:** Joel Zylberberg, Jon Cafaro, Maxwell Turner, Fred Rieke, Eric Shea-Brown

**Affiliations:** 1 Department of Applied Mathematics, University of Washington, Seattle, WA 98195, USA; 2 Department of Biology, Duke University, Durham, NC 27708, USA; 3 Department of Physiology and Biophysics, University of Washington, Seattle, WA 98195, USA; 4 Howard Hughes Medical Institute, University of Washington, Seattle, WA 98195, USA

## 

Neural activities are unreliable indicators of features of the external world [1], and an open question is how our nervous systems function robustly in the presence of this noise. One possibility arises from the observation that variability is often correlated between neurons [2], leading to the important theoretical question of when and how noise correlations affect neural population codes. Much work has investigated this issue, leading to impressive insights about how the relationship between the statistical structures of signals vs noise affects neural population coding. Despite this progress, an important issue has been largely overlooked by the field: that of firing-rate-dependent correlations. Notably, the same pair of neurons can display different noise correlations in response to different stimuli; those correlations coefficients typically increase with increases in the neurons' firing rates [2,3].

In this paper, we investigate the role of so-called "limited range" correlations on population codes, either in the presence, or the absence of rate-modulation of the correlation coefficients. Limited-range correlations are frequently-observed population-wide correlation structures in which cells with similar tuning curves have positive noise correlations, and the correlations decrease with decreasing tuning curve similarity [2,4]. These patterns of noise correlation are typically harmful to population coding [5] (yielding worse population coding performance than would be obtained with the same tuning curves and noise variances for all cells, but no noise correlations); these effects are somewhat dependent on the degree of heterogeneity in the population's tuning curves [6,7].

Experimentally reported noise correlations are usually averaged over stimuli, thereby masking any stimulus dependence. Herein, we will demonstrate that, when correlation coefficients increase with the product of mean neural firing rates (as in [2,3]), the stimulus-averaged correlation coefficients will display limited-range structure. When the rate dependence of these correlations is ignored, those correlations appear to be quite harmful to the population code, in accordance with previous theoretical work [5]. Surprisingly, when the rate dependence is taken into account, the correlations can yield much better population coding than would be obtained in the presence of uncorrelated noise. These effects persist for either homogeneous or heterogeneous sets of neural tuning curves. One prior study [8] also found that rate-dependent correlations can have very different impacts on population codes than can rate-independent ones, but did not make the connection between firing-rate-dependent correlations and the frequently observed limited-range correlation structure. Overall, our results emphasize that, for understanding the impact of limited-range correlations on neural population coding, the firing-rate dependence of those correlations is a potentially important consideration. Thus, it is important to report not only stimulus-averaged correlation coefficients, but also the relationship between those correlation coefficients and the neural firing rates.
